# How a discerning cytological examination can aid in the diagnosis of infectious diseases: case reports

**DOI:** 10.1590/1414-431X202010462

**Published:** 2021-01-08

**Authors:** D.K. Faria, J.N. de Almeida, C.S. Faria, B. Durante, B.F. Falasco, E. Terreri, L. Antonangelo

**Affiliations:** 1 Laboratório de Patologia Clínica, Departamento de Patologia, Hospital das Clinicas, Faculdade de Medicina, Universidade de São Paulo, São Paulo, SP, Brasil; 2 Laboratório de Investigação Médica, Hospital das Clínicas, Faculdade de Medicina, Universidade de São Paulo, São Paulo, SP, Brasil

**Keywords:** Cytology, Bronchoalveolar lavage, Pleural effusion, Ascites, Infectious diseases

## Abstract

Infections caused by uncommon and resistant pathogens in unusual sites have been increasingly reported in medical literature. We describe four cases of rare cytological findings and clinical impact for patients. In the first case, *Aspergillus* sp and *Pneumocystis jirovecii* were observed in the bronchoalveolar lavage of a patient with severe systemic lupus. In the second and third cases, we describe the presence of *Trichomonas* sp and *Strongyloides* sp larvae in samples of pleural and peritoneal fluid, respectively. The fourth report is about a patient with a wrist subcutaneous nodule whose synovial aspiration and cytology revealed the presence of brown septate hyphae. The early identification of the infectious agent in the cytological examination was essential for the introduction and/or re-adaptation of therapy in the four cases described. Patients in this report were immunocompromised with severe comorbidities, conditions often associated with unfavorable clinical outcomes.

## Introduction

The cytological examination of cavity fluids, sputum, and bronchoalveolar lavage (BAL) is part of the diagnostic routine of clinical laboratories, especially in tertiary referral hospitals. Although the exam focuses primarily on the analysis of cell predominance and oncotic cytology, the detection of bacteria, fungi, and parasites is part of the examination and the result is described in the medical report, aiding in the etiologic diagnosis of these unusual cases (1).

Infectious diseases, frequent in all areas of healthcare, are responsible for high rates of morbidity and mortality. For the correct therapeutic approach, the identification of the infectious agent by culture or PCR is the main goal in the clinical setting. However, the positivity of conventional Gram stain and culture in cavity fluids is generally less than 60% (2). Furthermore, with the increasing consumption of antimicrobial medications and the increasing at-risk population, especially of immunocompromised patients, infections caused by rare and resistant pathogens in unusual sites have been described more frequently in the medical literature (3).

Increased cancer incidence, the global spread of AIDS (acquired immunodeficiency syndrome), and the current availability of solid organ transplants have required a more thorough cytological examination of body fluids and BAL by clinical laboratories (4,5). Collection of these biological samples is not invasive and their analyses can provide results as efficient as those obtained from biopsies in a shorter time (within 24 to 48 h from collection).

In this report, we describe four cases of infrequent infectious agents found in different types of biological samples during routine cytological examination. In all cases, the cytological findings were essential for the etiological diagnosis and for therapeutic adequacy. The patients were immunocompromised with severe comorbidities, conditions frequently associated with unfavorable clinical outcomes.

## Description of cases

Four clinical cases from the Hospital das Clínicas, University of Sao Paulo Medical School, presenting unusual infectious agents in samples of body fluids are described. Samples were analyzed between January 2015 and September 2017 and included BAL (Case 1), pleural fluid (Case 2), peritoneal fluid (Case 3), and an aspirate from a wrist subcutaneous nodule (Case 4). Samples were submitted to routine cytological, biochemical (when relevant), and microbiological exams at the Clinical Laboratory, which is accredited by the College of American Pathologists (CAP). Clinical history, imaging exams, and other laboratory tests were used for case descriptions. The study was approved by the local Ethical Committee.

BAL was sampled in a siliconized conical tube; cavity fluids and nodule aspirate were collected in tubes coated with the EDTA anticoagulant. All samples were processed within 4 h from collection. After macroscopic analysis and counting of nucleated cells in a Neubauer chamber, the samples were cytocentrifuged (365 *g*, 25°C, 10 min) for slide preparation. Slides were stained with Leishman's hematological stain for routine cytology, and cytochemical staining was carried out to complement the etiological investigation with periodic acid-Schiff (PAS) reactive component, silver methenamine stain (Grocott), and Fontana-Masson stain.

### Case 1

**Figure 1:**
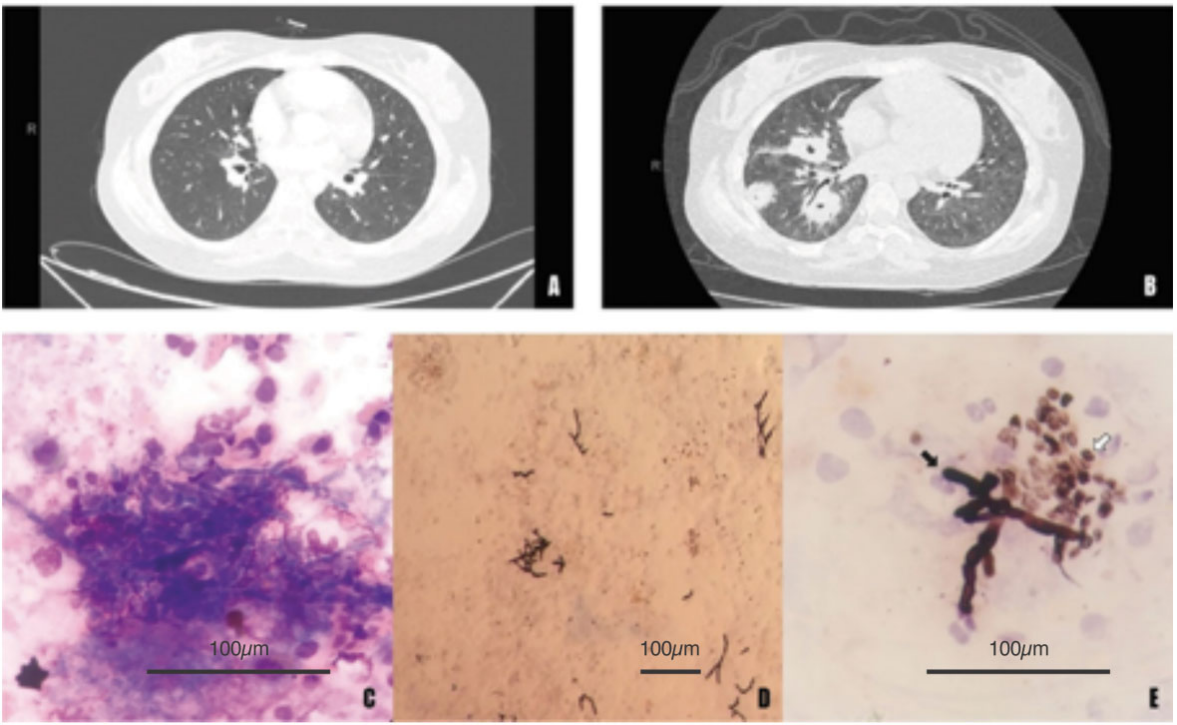
Aspects of chest tomography at admission (**A**) and after fifteen days of evolution (**B**) with the presence of excavated nodules with halo signal, initially considered to be of inflammatory/infectious etiology. **C,** Sample of bronchoalveolar lavage showing presence of macrophages, lymphocytes, neutrophils, and many hyphae and yeasts (Leishman staining, 600×). **D** and **E**, Presence of non-specific septate hyphae (black arrow) and yeasts (white arrow) suggestive of *Pneumocystis jirovecii* (silver methenamine staining, 200 and 600×, respectively). Scale bars: 100 μm.

A 22-year-old female with severe systemic lupus erythematous was hospitalized due to refractory thrombocytopenia and increased proteinuria. On admission, her main complaints were dyspnea and pain on the right hemithorax for the last fifteen days. Chest computed tomography revealed cavitated nodules on the right upper lobes (Figure 1A and B). The nodules were considered non-specific and of probable inflammatory/infectious etiology. The cytological analysis of the BAL showed a predominance of macrophages, a moderate number of lymphocytes and neutrophils, presence of septate hyphae, and yeasts suggestive of *Aspergillus* sp and *Pneumocystis jirovecii* (Figure 1C, D, and E). BAL culture identified growth of *Aspergillus* sp. Laboratory test for tuberculosis was negative and serology for human immunodeficiency virus (HIV) and for hepatitis B and C were non-reactive.

**Figure 2:**
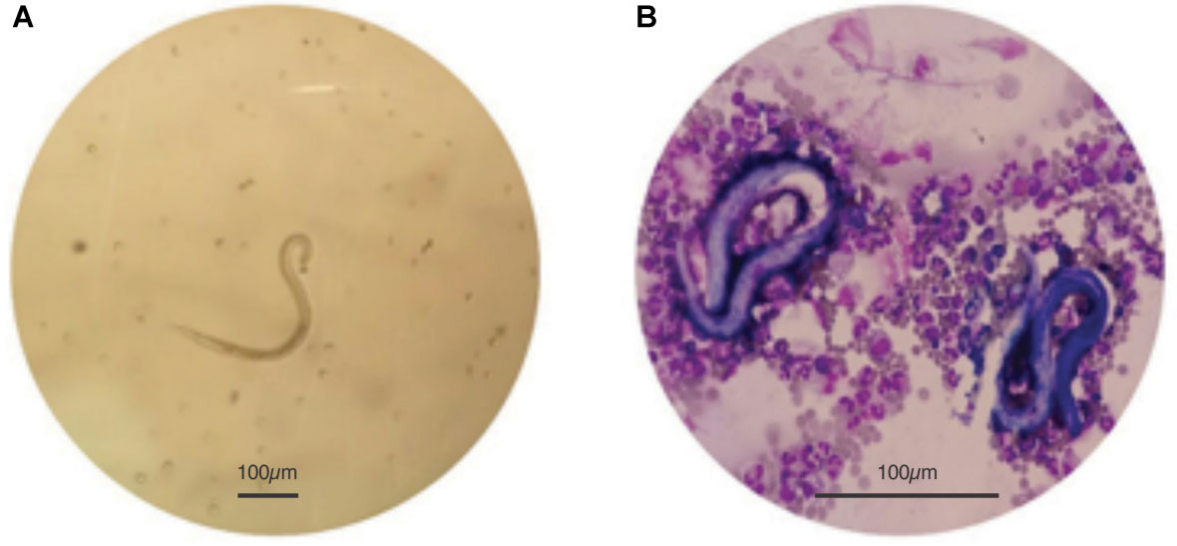
A, Presence of larvae in the fresh analysis of ascitic fluid (200×) and **B**, presence of *Strongyloides* larvae in a rich neutrophilic fluid (Leishman staining, 600×). Scale bars: 100 μm.

**Figure 3:**
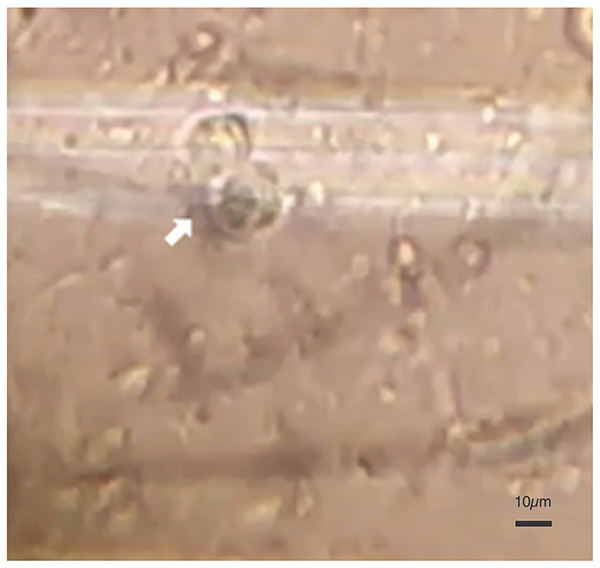
Pleural fluid fresh examination (600×, scale bar: 10 μm) showing flagellated forms of *Trichomonas* sp.

**Figure 4:**
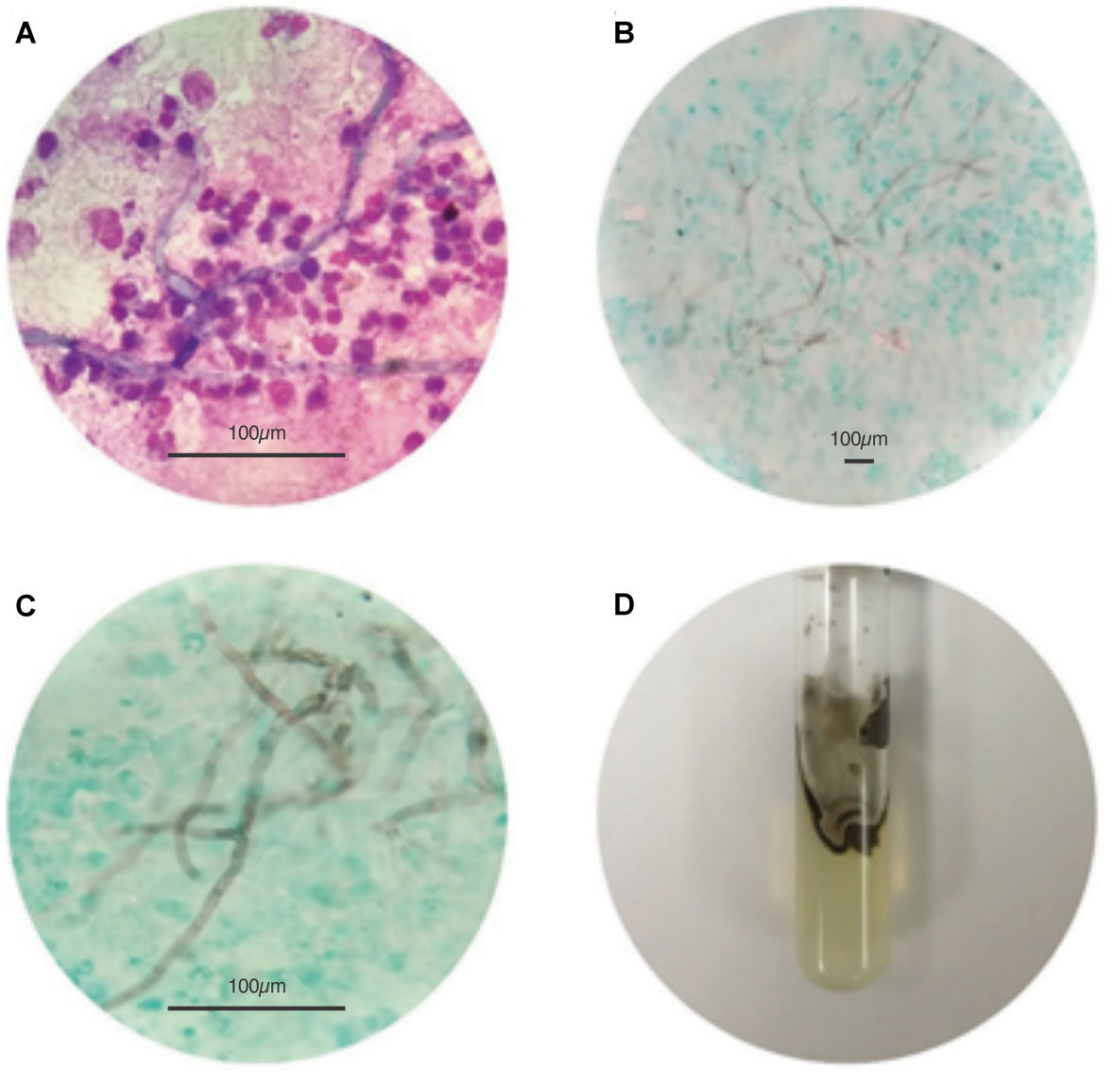
Presence of brown septate hyphae in the cytological examination of the cystic content with Leishman staining (600×) (**A**) and Fontana-Masson staining, 100× (**B**) and 600× (**C**), respectively. Scale bars: 100 μm. **D**, Aspect of fungal growth in Sabouraud agar medium.

**Table 1:** Literature data concerning the finding of *Strongyloides stercoralis* in peritoneal fluid.

Case report	Country	Gender (age in years)	Underlying condition	Clinical outcome
Avagnina et al., 1980 (18)	Argentina	Male (33)	Renal transplantation	Death
Hong et al., 2004 (19)	United States	Male (49)	HIV-positive	Favorable
Sav et al., 2009 (20)	Turkey	Male (67)	Peritoneal dialysis	Favorable
Saha et al., 2012 (21)	United States	Male (59)	Peritoneal dialysis	Favorable
Shukla et al., 2015 (22)	India	Male (24)	Diabetes and alcoholic	-
Zhang et al., 2017 (23)	China	Female (57)	Peritoneal dialysis	Death

HIV: human immunodeficiency virus.

#### Clinical follow-up

The patient was initially submitted to pulse-therapy with methylprednisolone, followed by immunoglobulin infusion, mycophenolate, hydroxychloroquine, danazol, amphotericin B, and sodium piperacillin-tazobactam. After the identification of *P. jirovecii* in BAL, sulfamethoxazole-trimethoprim was introduced. However, her respiratory functions worsened and evolved to metabolic acidosis. She was referred to the intensive care unit with orotracheal intubation and mechanical ventilation. Despite the therapeutic approaches, the patient died in six days.

### Case 2

A 55-year-old female was hospitalized due to a consumptive syndrome without defined etiology. She presented a history of dysphagia, weight loss (10 kg), and asthenia for 3 months, evolving with ascites and lower limb edema in the last month. The patient reported being an ex-smoker and ex-alcoholic, with no other previous diseases. On physical examination, the patient was in regular clinical condition, pale, anicteric, dehydrated, and emaciated (37.5 kg). Abdominal examination revealed bulging flanks suggestive of ascites. She had lower limb edema and oral moniliasis. The upper gastrointestinal endoscopy and biopsy showed moderate gastritis and severe bulboduodenitis, positive for *Helicobacter pylori.* Analysis of the ascitic fluid obtained by paracentesis revealed the presence of larvae suggestive of *Strongyloides* in a predominantly neutrophilic fluid (Figure 2). Parasitological examination of feces (methods of Faust and Lutz/Hoffman) confirmed eggs of *Strongyloides stercoralis* and in the modified Rugai method, the presence of *S. stercoralis* larvae. The duodenal biopsy showed intense inflammatory reaction and the presence of *Strongyloides* larvae.

#### Clinical follow-up

Patient progressed to renal failure, altered mental status, septic shock, and died a few days after admission.

### Case 3

A 46-year-old male, ex-smoker and ex-alcoholic, with a history of squamous cell carcinoma at the esophagogastric transition with liver, bones, and lymph nodes metastases, was hospitalized with complaints of dyspnea, secretory cough, and chest pain. The upper gastrointestinal endoscopy showed a stenotic infiltrative lesion at the esophagogastric transition. Thoracic/abdominal tomography and positron emission tomography (PET) revealed several mediastinal lymph node enlargements (up to 4.2 cm) in the transverse axis and parietal thickening of the esophagogastric transition, the liver with small hypodense nodules in segments II and V, a solid and heterogeneous retroperitoneal mass medially to the small gastric curvature (6.7×4.7 cm), and right pleural effusion. The patient was diagnosed with pneumonia, complicated pleural effusion, and superior vena cava compression syndrome. Antibiotic therapy with piperacillin-tazobactam and vancomycin and full anticoagulation were introduced. Thoracentesis was performed and analysis of the pleural fluid showed flagellate elements suggestive of *Trichomonas* sp (Figure 3).

#### Clinical follow-up

Despite metronidazole therapy, his clinical condition worsened and he died. Unfortunately, there was a limitation for the identification of the species of *Trichomonas* sp.

### Case 4

A 60-year-old male rural worker, ex-smoker, with a previous history of hypothyroidism, epilepsy, chronic renal failure, and cardiac transplantation due to dilated Chagas cardiomyopathy, sought medical assistance due to an increasing cyst on the left wrist. The patient was using immunosuppressive therapy (mycophenolate sodium, cyclosporin, and prednisone). On examination, the cyst was mobile, well-delimited, painful, and without phlogistic signs. Cytological analysis of the cystic fluid revealed moderate cellularity with a predominance neutrophils and macrophages and the presence of many brown septate hyphae. Fontana-Masson staining showed melanin-positive hyphae (Figure 4A, B, and C); the mycological culture confirmed the growth of melanized fungal colonies, with hyphae and spore in Sabouraud agar medium (Figure 4D). The ultrasound-guided biopsy revealed a chronic inflammatory process with foci of suppurative acute activity and the presence of hyphae and fungal spores, confirming the cytological findings.

#### Clinical follow-up

After the laboratory results, itraconazole was introduced to the patient and he is currently controlled and in outpatient follow-up.

## Discussion

### Co-infection of *Aspergillus* sp and *Pneumocystis jirovecii* in BAL sample

*Aspergillus* sp, mainly *Aspergillus fumigatus*, is a ubiquitous saprophytic fungus transmitted by inhalation of spores or conidia. The mycosis can be manifested in several clinical forms: invasive, chronic necrotizing, allergic bronchopulmonary, and aspergilloma (6). Invasive aspergillosis is a frequent cause of death in immunocompromised patients, especially in those with hematological disorders (7). Currently, the diagnosis based on biomarkers (galactomannan, polymerase chain reaction, and β-D-glucan) has significantly improved cytological diagnosis (8–10).

*P. jirovecii* is a yeast fungus that colonizes the lungs, triggering pneumonia in immunocompromised patients, especially in those HIV-infected with T-helper (CD4 +) cell count lesser than 200 cells/mm^3^ (11).

The co-infection of *A. fumigatus* and *P. jirovecii* in HIV-negative patients is a rare event. A recently published systematic review identified only 7 cases in the medical literature (12). To date, this is the first case of *Aspergillus* and *P. jirovecii* co-infection identified in a bronchoalveolar lavage sample in Brazil.

### Identification of </emph>*Strongyloides* sp in ascites

Strongyloidiasis is a parasitic infection caused by *S. stercoralis* or *S. fuelleborni*, a nematode widely distributed in the world, especially in tropical areas (13). The diagnosis is usually based on the direct visualization of larvae in feces, with low sensitivity. Enzyme-linked immunosorbent assay, western blot, and molecular techniques can be used as complementary methods (14). In adults, the main risk factors associated to strongyloidiasis are related to impairment of the immune system, caused by alcoholism, use of corticoids, neoplasms, HIV infection, and human T-lymphotropic virus 1 (13,15).

The life cycle of *S. stercoralis* alternates between generations of free-living and parasite forms and the host becomes infected through the skin by the filarial larva, initiating an intestinal and pulmonary cycle. In the parthenogenic stage, the nematode deposits eggs in the intestinal mucosa, which hatch and release rhabditoid larvae that develop or mate in the soil or directly self-infect the host through the terminal intestinal mucosa (13,16). Infected patients can be asymptomatic or present cutaneous, gastrointestinal (diarrhea, abdominal discomfort, nausea, anorexia), and/or pulmonary symptoms, or can even present more severe symptoms of hyperinfection (17 [Bibr B18] [Bibr B19] [Bibr B20] [Bibr B21] [Bibr B22] [Bibr B23]). The finding of *S. stercoralis* in peritoneal fluid is a rare event, with only 6 cases reported in a recent literature review (Table 1).

### *Trichomonas* sp in pleural fluid

*Trichomonas* sp is a group of flagellate protozoa, with four species infecting humans: *Trichomonas vaginalis, Dientamoeba fragilis, Pentatrichomonas hominis*, and *Trichomonas tenax* (24). The infection caused by *Trichomonas vaginalis* is a common sexually transmitted disease, with 280 million new cases worldwide. Patients are usually asymptomatic (50-70%), although women may present vaginal pruritus and discharge, vulvovaginitis with erosive lesions, and edema with hemorrhagic areas on the cervical wall, increasing the risk for concomitant HIV infection (25,26). *P. hominis* (formerly called *Trichomonas hominis* or *Trichomonas intestinalis*) lives by commensalism in the intestine, usually causing asymptomatic infection; however, it can cause diarrhea in immunocompromised patients (24). *D. fragilis* has been associated with recurrent abdominal pain in children, irritable bowel syndrome in adults, and increased risk of intestinal infections in patients using antibiotics (27). *T. tenax* is associated with oral cavity infections. In a systematic review of 47 studies, the presence of gingivitis and periodontitis ranged from 0 to 94.1% (28).

Pulmonary infection by *Trichomonas* sp is an uncommon event, more associated with *T. tenax* (29). According to Leterrier et al. (30), only 17 cases of *Trichomonas* sp were identified in pleural fluid according to the medical literature since 1965. In Brazil, we did not find any previous report.

### Phaeohyphomycosis in wrist nodule

Phaeohyphomycosis is an opportunistic fungal infection caused by filamentous species that have brown melanin pigments in their cell walls (31,32). It is an uncommon cause of disease in humans, although it can cause infections in immunocompromised individuals (32). Epidemiological data are scarce in the medical literature. Rees et al. (33) reported the incidence of 1:1,000,000 cases per year in San Francisco, in the United States. Cutaneous or subcutaneous nodules are the most common form of the infection, which presents an indolent course (34). The infection results from the inoculation of the fungal agent through the skin by minor trauma when in contact with contaminated soil, plants, or wood (33,34). Revankar et al. (35) reported episodes of fever (76%), pulmonary infections (46%), cutaneous manifestations (33%), such as rashes and ulcers, cardiac infections (29%), central nervous system infections (22%), and urinary infections (22%) in 72 patients studied. Some cases of scrotal pouch infection have been reported (36–38). In our report, the identification of the black fungus excluded the suspicion of wrist neoplasia and provided the basis for the adequate surgical treatment with patient clinical improvement.

Regarding the complexity of the reported cases, the results of the cytological examination were released in less than 48 h, and patient management was complemented/reconsidered after medical contact. Most clinical laboratories report only the leukocyte differential in the cytological examination of cavity fluids/BAL, referring microbiological, molecular, and oncotic cytology to Microbiology, Molecular Biology, and Pathological Anatomy laboratories, delaying the release of exam results.

The results of fungal or mycobacterial culture take days or months, and not all laboratories have a wide molecular menu in their routines; thus, we reinforce that the cytological examination should be quantitative (total nucleated cells and respective percentages), with research of anomalous elements, such as fungi, bacteria, tumor cells, etc. The double centrifugation used in the preparation of the slides favors the finding of rarer elements, allowing the presumptive diagnosis generally in 24-48 h. The immediate communication of the cytological findings can result in therapeutic adequacy, which is often fundamental to the patient's clinical outcome.

### Final remarks

In the present study, we described a series of cases whose etiological diagnoses were triggered by routine cytological findings. In all cases, identification of the infectious agent was essential for the introduction and/or re-adaptation of therapy. The cases were related to immunocompromised patients or patients with severe comorbidities, conditions often associated with unfavorable clinical outcomes.
